# Prospects and strategies for malaria elimination in the Greater Mekong Sub-region: a qualitative study

**DOI:** 10.1186/s12936-019-2835-6

**Published:** 2019-06-20

**Authors:** Nils Kaehler, Bipin Adhikari, Phaik Yeong Cheah, Lorenz von Seidlein, Nicholas P. J. Day, Daniel H. Paris, Marcel Tanner, Christopher Pell

**Affiliations:** 1 Swiss Tropical and Public Health Institute, Basel, Switzerland; 2 University of Basel, 4051 Basel, Switzerland; 3 Mahidol Oxford Research Unit, Faculty of Tropical Medicine, Mahidol University, Bangkok, Thailand; 4 Centre for Tropical Medicine and Global Health, Nuffield Department of Medicine, University of Oxford, Oxford, OX3 7FZ UK; 5 The Ethox Centre, Nuffield Department of Population Health, University of Oxford, Old Road Campus, Oxford, OX3 7LF UK; 6 Amsterdam Institute for Global Health and Development (AIGHD), Amsterdam, The Netherlands; 7 Centre for Social Science and Global Health, University of Amsterdam, Amsterdam, The Netherlands

**Keywords:** Malaria control, Malaria elimination, Health systems, Policy, Targeted approach, Surveillance and response, Elimination strategies, Governance

## Abstract

**Background:**

As malaria elimination becomes a goal in malaria-endemic nations, questions of feasibility become critical. This article explores the potential challenges associated with this goal and future strategies for malaria elimination in the Greater Mekong Sub-region.

**Methods:**

Thirty-two semi-structured interviews were conducted with policy makers (n = 17) and principal investigators (n = 15) selected based on their involvement in malaria prevention, control and elimination in the GMS. Interviews were audio-recorded and transcribed for qualitative content (thematic) analysis using QSR NVivo.

**Results:**

All respondents described current malaria control and elimination strategies, such as case detection and management, prevention and strengthening of surveillance systems as critical and of equal priority. Aware of the emergence of multi-drug resistance in the GMS, researchers and policy makers outlined the need for additional elimination tools. As opposed to a centralized strategy, more targeted and tailored approaches to elimination were recommended. These included targeting endemic areas, consideration for local epidemiology and malaria species, and strengthening the peripheral health system. A decline in malaria transmission could lead to complacency amongst funders and policy makers resulting in a reduction or discontinuation of support for malaria elimination. Strong commitment of policymakers combined with strict monitoring and supervision by funders were considered pivotal to successful elimination programmes.

**Conclusion:**

Against a backdrop of increasing anti-malarial resistance and decreasing choices of anti-malarial regimens, policy makers and researchers stressed the urgency of finding new malaria elimination strategies. There was consensus that multi-pronged strategies and approaches are needed, that no single potential tool/strategy can be appropriate to all settings. Hence there is a need to customize malaria control and elimination strategies based on the better surveillance data.

## Background

### The burden of malaria in the Greater Mekong Sub-region

In line with global trends, the Greater Mekong Sub-region (GMS) has recently recorded declines in malaria-related mortality and morbidity, which reflect a substantial reduction in transmission [1–4]. The recent emergence of artemisinin resistance in the region could however reverse these gains [5, 6]. Scientists are concerned about the potential spread of artemisinin-resistant *Plasmodium falciparum* parasites to Africa, where this would likely result in a major public health crisis [7]. With the apparent decrease in regional malaria prevalence and the need to contain the spread of resistance, researchers have redoubled efforts and adapted strategies to eliminate malaria from the GMS [8, 9].

### Challenges of malaria elimination in the Greater Mekong Sub-region

Efforts to contain the spread of artemisinin-resistant malaria must overcome several challenges [10]. In the GMS, malaria transmission now mostly occurs in remote locations, such as forest fringes and forested areas, and among populations who live and depend on the forest, including- migrant workers and subsistence farmers [11]. Access to these areas to deliver malaria control and prevention programmes is therefore difficult. Additional challenges include the increasing resistance to insecticides, including pyrethroids and DDTs [12], and the wide circulation of substandard and counterfeit antimalarials in the region [13–15].

Health systems are often weak in the region, with limitations in terms of implementing proven interventions, poor governance, corruption and weak supply chain logistics [16, 17]. Malaria case detection and treatment depends on the peripheral health system and village malaria workers (VMWs) or village health volunteers [18–20]. Village malaria workers diagnose malaria and provide treatment to community members, in addition to maintaining malaria surveillance data [18, 20]. Irregular funding for VMWs and inadequate opportunities for their skill development and training have had a negative impact on treatment and the quality of surveillance data [21].

### Current malaria elimination strategies in the Greater Mekong Sub-region

Multi-pronged approaches are necessary to tackle these challenges and several elimination strategies have recently been piloted. For example, targeted malaria elimination (TME), which includes mass drug administration (MDA), the distribution of insecticide-treated bed nets (ITNs), strengthening of village networks of health workers, has been assessed in Cambodia, Lao PDR, Myanmar and Vietnam [8, 22–29]. The recent scale-up of TME along the Thai–Myanmar border, which included strengthened, diagnosis and treatment with additional malaria posts established within the villages, has illustrated how timely access to treatment, together with targeted MDA, can help reduce the incidence of *P. falciparum* to zero [9]. These findings suggest that elimination in the GMS could become a reality [30].

### Malaria elimination as a goal in the Greater Mekong Sub-region

Nations within the GMS have adopted the goal of eliminating all forms of malaria by 2030 [31] (see Table 1 for details) [32, 33]. The World Health Organization’s Global Technical Strategy 2016–2030 (GTS) identifies three essential pillars that underpin strategies for malaria elimination [33]: (1) ensuring universal access to malaria prevention, diagnosis and treatment; (2) accelerating efforts towards elimination and attainment of malaria free status; and, (3) transforming malaria surveillance into a core intervention. The pillars can be tailored to national and sub-national settings to increase the effectiveness of elimination programmes [33]. The GTS together with the WHO’s Action and Investment to Defeat Malaria 2016–2030 (AIM) lay out a detailed pathway to elimination.

**Table 1:** Context and prospect of malaria elimination in the Greater Mekong Sub-region

	Cambodia (ref. National Strategic Plan for Elimination of Malaria (2011–2025) [75]	Laos (ref. National Strategic Plan for Malaria Control and Elimination 2016–2020) [76]	Myanmar (ref. National Plan for Malaria Elimination in Myanmar 2016–2030 (NMCP) [77]	Vietnam (ref. National malaria control program Vietnam) [78]	Thailand (ref. National malaria control program of Thailand) [79]
Aims	Eliminate artemisinin-resistant parasites of falciparum malaria by 2015 Achieve zero deaths by falciparum malaria by 2020 Eliminate all forms of malaria in Cambodia by 2025	Eliminate malaria in Lao by the year 2030 Establish a tight collaboration between all governmental departments and ministries as well as collaborating with external (implementing-) partners to strengthen the health system at all levels	Interrupt malaria-transmission and eliminate malaria throughout the entire country by 2030 Maintain a malaria-free status in regions where transmission has been interrupted and prevent re-establishment of local transmission Establishing universal access to malaria detection- and treatment Close coordination with communities, national- as well as international NGOs, agencies of the UN and other financial stakeholders and partners	Achieve malaria elimination by 2030	Eliminate malaria by 2024
Main interventions	Promotion of use of ITNs Provision of comprehensive services for free malaria-diagnostic and treatment Stop spread of malaria-drug resistant parasites Control of sale of anti-malarials to reduce the spread of ineffective and/or fake-drugs MDA in selected areas within Cambodia Development and implementation of technical- and operational plans for elimination of malaria with both national- and international partners	NSP 2016–2020 is the first part of a three-phase plan to eliminate all forms of malaria in Cambodia It focusses primarily on the impact-reduction of multi-drug resistance in the southern parts of the country, while promoting efforts to eliminate malaria in the in northern- and central parts of Lao The second phase focusses on the elimination of falciparum malaria in the entire country while eliminating all forms of malaria in the northern provinces The third phase focusses on the elimination of all forms of malaria in the entire country by 2030	Key interventions Case management Disease prevention Malaria surveillance and prevention Control of malaria outbreaks	Control of malaria in moderate and high malaria endemic areas where the targets for 2020 are morbidity below 0.15 per 1000 population, mortality below 0.02 per 100,000 population and malaria elimination in at least 40 provinces (targets have already been achieved)	One of the key component of national malaria elimination is the 1-3-7 strategy Real-time notification investigation and response parameter for individual cases as they are detected diagnostics and medicines available at provincial level (health facilities) Cross + border collaboration with neighbouring countries as around 80% of malaria cases among migrant- + and mobile populations, clusters concentrated along Cambodian- and Thai–Myanmar border
Funding	Total costs estimated: US$755,318,886 Budget Cambodian Government: US$131,740,244 Total budget gap: US$623,578,643 (has to be covered by external funding)	Total costs estimated: US$72,800,000	Total costs estimated: US$1,323,000,000–1,403,000,000	Total costs estimated: US$75,000,000 Budget from two sources: State budget (28 mil.) and international supported projects (50 mil. World Bank loans and Global fund) 55–80% of malaria-related budget comes from Global Fund in malaria-endemic districts	Total costs estimated: US$64.800,000 (2015–2021)

### Aim

Adopting the goal of elimination by 2030 without examining potential barriers may lead to wasted resources and may ultimately contribute to failure. Policy makers and leading malariologists can offer insights into the practicalities of elimination, which have seen insufficient attention. Drawing on semi-structured interviews, this article explores the perspectives of stakeholders working in malaria elimination at the policy level (governmental and non-governmental) and research, with a view to addressing the following research questions:

1. What do researchers, policy makers and funders perceive to be major barriers, in terms of practicality and feasibility, to achieving malaria elimination in the GMS?
2. What do they perceive as plausible strategies to overcome such barriers?

## Methods

Data were collected as part of the qualitative study, “Stakeholder perspectives on mass drug administration and malaria elimination in the Greater Mekong Sub-region”.

### Respondents

Respondents were recruited based on their expertise (principal investigators of current malaria elimination studies and senior malariologists) or decision-making roles in malaria prevention, control and elimination in the GMS (policy makers in Thailand, Cambodia, Myanmar, Vietnam or Laos, and funders, such as from the World Health Organization and the Bill and Melinda Gates Foundation). Potential respondents were identified through a combination of (1) a snowball approach, and (2) bibliography and web searches. The appropriateness of potential respondents, identified during interviews or web searches, was discussed among core members of the research team.

The contact details of potential respondents were obtained from institutional websites or from other respondents. Potential respondents were subsequently contacted by email. None of the potential respondents explicitly refused to participate. Two potential participants could not be reached for the interview.

The recruitment of respondents was based on purposeful sampling and recruitment continued until theoretical saturation (whereby newly collected information does not provide additional insights) [34, 35]. To include maximum diversity of opinion among respondents, respondents from the field of malaria research and policy were approached who were recognized as endorsing different approaches to malaria elimination in the GMS.

### Study tool

A topic guide for the semi-structured interviews was developed in consultation with members of the study team, drawing on the initial research questions. Topics included malaria elimination, MDA and community engagement. Under these broad topics, a flexible and iterative approach was taken to questioning to elicit in-depth information and to ensure that relevant topics were not neglected. This study aimed to explore perspectives of researchers, policy makers and funders concerning the practicality and feasibility including challenges of achieving malaria elimination in the GMS without specific focus on species of malaria.

### Data collection

Data collection was conducted between October 2016 and April 2017 at various locations in Thailand, Myanmar, Cambodia, Laos, Vietnam and the USA. Whenever possible, interviews were conducted face-to-face at a study site of a pilot malaria elimination strategy, selected international tropical medicine conferences or at the ministerial offices. If a face-to-face meeting was not possible, Skype or telephone interviews were conducted. Two respondents were not available for a face-to-face or a telephone/Skype-interview but responded to an email questionnaire.

Interviewers were conducted by the first author, a medical doctor, with master’s in tropical medicine and public health, training in qualitative and quantitative social science research methods. Data collection (and analysis) was supervised by an experienced qualitative social scientist (last author). All interviews were conducted in English and ranged from 20 to 90 min in length. On average, interviews with policy makers and funders were longer than those with scientists. Interviews were audio-recorded and transcribed verbatim by an independent transcriber and, using the recordings, the interviewer checked all transcripts for accuracy.

### Data analysis

The interview transcripts were analysed using qualitative data analysis software (NVivo 11; QRS International, Australia). A codebook adapted from previous qualitative research on TME and community engagement (in Cambodia, Laos and Myanmar) was used [26]. Line-by-line coding of transcripts used pre-established themes (deductive approach), e.g., malaria elimination, community engagement, and MDA, followed by themes that emerged during the data analysis (inductive approach). Analysis continued by identifying and explaining prominent themes and patterns amongst respondents and outliers. For coding reliability, all coding was initially done independently by three researchers (NK, BA and CP) and then compared and discussed. Any disagreement among the researchers was resolved by discussions and only the agreed on codes were finally presented.

### Ethics approval

Initial data collection was conducted as part of the ethics approval for the TME project [8, 26] that included ethical approval from each countries and are as follows:

Cambodia: National Ethics Committee for Health Research Cambodia (NECHR 0042 & 0051) and the Oxford Tropical Research Ethics Committee (OXTREC; 1017–13); Laos: Lao National Ethics Committee for Health Research (Ref. No. 013-2015/NECHR), Government of the Lao PDR and the Oxford Tropical Research Ethics Committee (1015–13); Vietnam: the Institute of Malariology, Parasitology and Entomology in Ho Chi Minh City (185/HDDD), the Institute of Malariology, Parasitology and Entomology in Qui Nhon and the Oxford Tropical Research Ethics Committee (1015-13); Myanmar: ethics Review Committee of the Department of Medical Research (Ref: 74/Ethics 2014) and the Oxford Tropical Research Ethics Committee (23–15; 1015–13), the Tak Province Community Ethics Advisory Board and the village committees.

Subsequently, further Ethical approval was obtained from the Oxford Tropical Research Ethics Committee (OxTREC) and was approved on 31 January, 2017 (Unique Protocol ID: OxTREC ref: 5122-16). Verbal informed consent was obtained from all respondents prior to interviews. Interviewees were given an explanation about the study, its rationale and the voluntary nature of their participation. All respondents were briefed that they could drop out of the study at any time during the interview without providing justification. Anonymity and confidentiality were secured for all the interviewees.

## Results

Between October 2016 and April 2017, a total of 32 semi-structured interviews (SSIs) were conducted with 17 policy makers and 15 leading malariologists. None of the respondents explicitly refused to participate but two were not responsive to repeated e-mails. Policy makers included three women and 14 men; researchers included three woman and 12 men.

Guided by the research questions, the findings are presented by the themes that emerged during the interviews, integrating responses from policy makers and researchers. The themes included: (1) tools for malaria elimination; (2) feasibility of malaria elimination; and, (3) future strategies for malaria elimination.

### Tools for malaria elimination

Most of the broad strategies for malaria elimination described by policy makers reflected national malaria policies. Interventions, such as detecting and treating malaria cases as well as the importance of a strong surveillance system, were highlighted by most researchers.

*“Actually, we’re focusing on five main pillars: malaria prevention, vector control, case management, detecting and treating the cases, and improving the surveillance system”* (SSI with a policy maker #3).

#### Village malaria workers

Drawing on the recent experience in the Thai–Myanmar border region, researchers particularly supported the establishment of malaria posts to support elimination. However, several respondents were skeptical about the sustainability of health workers in the villages.

*“[…] Now that we have the data, we know that the malaria posts are pretty easy, scalability*-*wise, and they have a huge impact [on malaria incidence].”* (SSI with a senior researcher #26)

*“Everybody likes talking about community health workers, village health workers. Give me one place where government has scaled this up and maintained it over a long period of time while making sure that these people are supervised, trained, supplied so that they get regular supplies.*” (SSI with a policy maker #14)

#### Insecticide-treated bed nets

Several researchers questioned the benefits of bed nets for malaria control in the GMS. They highlighted the biting habits of the highly diverse vectors in the region, including *Anopheles maculatus, Anopheles minimus, Anopheles dirus*, which generally bite outdoors and before dusk. This contrasts with sub-Saharan Africa, where the principal vectors, specifically *Anopheles gambiae*, bite indoors and at night. Several researchers described bed nets as insufficient to prevent human-vector contact in the GMS and as a waste of money.

*“We confirmed that bed nets are utterly useless. We confirmed that. We proved it. Nobody wants to listen to this because bed net is a dogma. You have to have a bed net. If you don’t have a bed net; if you don’t distribute bed nets to people, you are a criminal. They are utterly useless and they are expensive.”* (SSI with a senior researcher #27)

*“Bed nets work in most places but don’t work well in the Southeast Asian region because, again, of the behaviour of the vector and the early evening biting patterns and also the behavior of the humans.”* (SSI with a senior researcher #31).

Meanwhile, most policy makers in the GMS reported that bed nets are a suitable and important tool for malaria control.

*“We are using the current tools, like bed nets and hammocks for preventing malaria, and also repellent”* (SSI with a senior policy maker #3)

#### Case detection and management

Most researchers agreed on the importance of effective case-detection and management systems and highlighted especially the establishment and expansion of well-functioning health and/or malaria posts in affected areas.

*“But just as the logistics rolled out and everything, it dawned on us that the malaria posts were going to be more and more important. And now that we have the data, we know that the malaria posts are pretty easy, scalability*-*wise, and they have a huge impact* “(SSI with a senior researcher #26).

#### Mass drug administration

MDA was mentioned by some researchers and they were aware of its potential benefits, especially in high transmission areas. A few researchers described MDA as playing an important role in elimination in general; most considered it to have an adjunctive function to accelerate the process within a package of interventions. All respondents considered relying solely on MDA questionable. Researchers identified specific challenges related to MDA, its implementation, and the need to achieve high coverage, especially with several intervention rounds. The logistics of MDA were also considered expensive.

*“First of all, MDA cannot lead to elimination. It is now recognized that MDA must be part of the package*—*if you want to*—*for elimination. This would be part of good malaria case management and vector control as to give good case management, good diagnosis, good treatment and eventually distribute nets or hammocks, if it’s useful.”* (SSI with a senior policy maker #5).

*“Now on the specific question of MDA, I would say two things. First is that it’s not the most important tool for malaria elimination by far; with that drug and that schedule and under those circumstances, it’s not the most important tool of elimination but it helps. What it does is that it accelerates elimination. You will eliminate malaria without doing it, no doubt about it. We have done it in refugee camps in Thai villages, we can. It just takes longer. just takes longer.”* (SSI with a senior researcher #27).

#### Vaccination

Several researchers mentioned the potential role of vaccinations, but none of the interviewees was aware of a malaria vaccine that could be used on a large scale. In addition, many showed concern regarding ‘safety and efficacy’ of currently available or tested vaccines.

*“So I mean, if we had a vaccine that was safe and effective and deployable, affordable, and protected people for a year, that’d be great, and maybe we have got one already, and we need to evaluate it, but for me, in this region, we just have to rely on those drugs, and we’re losing those drugs.”* (SSI with a senior researcher #31).

### Challenges of malaria elimination

Among the policy makers, a few suggested large-scale elimination interventions when proposing possible future malaria elimination strategies, referring to successful interventions in China and Sri Lanka,

*“The operational feasibility*—*is more complicated and is where I argue don’t try to contain in small pockets; try to eliminate the whole region through regionally coordinated efforts.”* (SSI with a policy maker #14)

Policy makers also pointed out significant challenges of implementation: most favoured targeting malaria in particular endemic areas with high-risk populations, such as mobile migrants and forest goers.

*“The next step for malaria elimination is we have to work hard, we have to intervene in various affected areas. We cannot neglect the wild areas… In this country, we try to focus on where the problem is.”* (SSI with a policy maker #9)

### Funding

Researchers and policy makers mentioned the importance of continuous funding to reach the goal of eliminating malaria. Several respondents highlighted the important role of the Bill & Melinda Gates Foundation and the Wellcome Trust. Significant concerns regarding future funding were highlighted by several government representatives, warning that malaria elimination may fail without continuous financial support.

*“But still we need more money. If we had the money we also can invest with our guidelines to implement our strategic activity.”* (SSI with a policy maker #4)

Among the researchers there was skepticism about whether funding was a big problem.

*“What if there is too much money with too little governance. If we really wanted to eliminate malaria, it would be rather expensive. It depends on how fast you want to go. But to go fast would be*—*I mean, to do mass drug administration on an extensive scale in the Greater Mekong sub Region would cost a lot of money, no doubt. But what happens at the moment because the governance is so weak, and the donors are so irresponsible about the money they provide.”* (SSI with a senior researcher # 31)

Some described how funding has led to significant corruption in the system, which may threaten elimination efforts and consequently lead to further spread of resistant malaria.

*“There is too much money. Now in Southeast Asia for eliminating malaria, there is too much money. That creates corruption. Corruption is going to kill it because the work will not be done, malaria will not be eliminated and it will come back, resistant this time. There is too much money so people are corrupt and there’s corruption everywhere. When malaria comes back; not only will there not be more money probably, but it will be too late.”* (SSI with a senior researcher #27).

Some researchers blamed many policy makers’ poor knowledge of malaria as a threat to achieving elimination. They pointed out that several strategies, based on unfounded assumptions, had been implemented and failed to have a positive impact but consumed a lot of money.

*“The big corruption is in the brain, it is that if people are convinced- and there are honest people in the Ministries. They are honest. They are not corrupt. But if they think that they can utilize this money all the way up here without spending money here, I call it corruption, corruption of the mind. That corruption of the mind is not only at the Ministry of Health, it’s all the way to the WHO. The WHO thinks that if you distribute bed nets to these people, you are going to eliminate malaria. That’s corrupted because it’s wrong thinking. It’s the corruption of the system. Your operating system is corrupted. Your computer is corrupted. You have corrupted files in your operating system and that’s a problem. You have to delete those files. As long as you don’t do that, it doesn’t work.”* (SSI with a senior researcher #27).

Some suggested that spending money on inefficient strategies results in a decreased budget for necessary future interventions. Other researchers described how the irresponsibility of donors and inadequate supervision of funding leads to ‘a disconnect’ between the different levels of programme administration, from the donor down to the interventions in the field. Researchers described the acceptance of dishonesty and corruption as critical factors that might undermine malaria elimination efforts. Other respondents viewed the ineffectiveness of programmes as intentional and a way of maintaining funding streams.

*“Inefficiency is a nice word. Corruption is a nasty word. They merge beautifully into each other.”* (SSI with a senior researcher #31).

Researchers emphasized the importance of lasting and effective surveillance system, which is difficult to sustain with just volunteer workers.

*“I think what will happen is they* [the governmental institutions] *will rely on volunteers and that it will work for a period of time, and then after a while, people will get paid jobs, and so they move away or something falls apart. And it doesn’t end up working.”* (SSI with a senior researcher #26).

### Future strategies for malaria elimination

Researchers and policy makers were generally optimistic about the possibility of eliminating malaria and referred to the recent commitment and endorsement by most GMS countries and the WHO. However, some researchers expressed concern that, due to the heterogeneity of malaria, individual strategies will have to be tailored to cope with the diversity of the region, and that includes local epidemiology, species, geographic situation, accessibility, willingness and support of the local population to accept the particular intervention.

*“The feasibility right now if you allow me to refer to Laos. One of the main challenges for elimination is capacity to de*-*strategize and to target interventions that are more efficient and more applicable for elimination but it’s not only because of the capacity of the country but also because there is different*—*it’s quite a heterogeneous situation of malaria in the country in the south with transmission of malaria both vivax and falciparum.”* (SSI with a policy maker #1).

Referring to political commitment, researchers and policy makers pointed out the danger of a centralized strategy of malaria elimination, which may fail due to a lack of expertise and ignorance of the situation in endemic areas, which could threaten the success of elimination. Understanding and engaging with communities where transmission is ongoing was emphasized as an important strategy.

*“The way they monitor now and operate, that is a very centralized power in one person or in the whole effort. In the capital, one person makes promises and the person decides 100% by himself who to hire. To me it’s not very efficient way of working. Especially when you have to work at community, at grass root*-*level, and also because at all the levels of the health system people have no information or have not been trained on elimination.”* (SSI with a policy maker #1).

Some researchers mentioned that although it may be possible to eliminate *P. falciparum*, the challenge of completely eliminating malaria depends on addressing vivax malaria.

*“If we get to real elimination, particularly in Asia and Latin America, we have got to kill vivax.”* (SSI with a senior researcher #20).

The elimination of all malaria species was considered possible by some researchers, but requires recognizing the importance of intense vector control, community engagement and MDA.

*“Then [in] 2000 we report[ed] it: malaria eradication is possible on islands by existing tools that are there. MDA and sustained vector control with [a] high degree of community engagement.”* (SSI with a senior researcher #21).

In addition to strengthening the existing case detection and management programmes at village level, researchers described targeting large communities with multiple strategies as critical to accelerate malaria elimination. There were clear differences between researchers and policy makers regarding approaches to halt the spread of artemisinin combination therapy resistance from the region. Policy makers emphasized the importance of a well-functioning primary health care system, whereas researchers focused on the acceleration of malaria elimination by using more intensive approaches such as MDA and mass screening and treatment, with a focus on specific populations, such as forest goers and migrants.

## Discussion

### Overall findings

Policy makers and researchers agreed regarding the urgency of malaria elimination against the backdrop of increasing anti-malarial resistance (in the GMS). Researchers and policy makers expressed concern over the currently available tools for malaria elimination, challenges related to implementation and future strategies. Although policy makers and researchers agreed that there was a need to strengthen case detection and treatment programmes, opinions differed in their assessment of the efficacy of the available elimination tools and strategies. Researchers and policy makers agreed on the absence of and the need for an omnipotent tool, e.g., vaccine that provides complete, life-long protection and could be used globally. They also recommended a tailored approach targeting primarily malaria endemic settings or pockets of malaria in combination with intense community engagement and collaborative efforts to strengthen the peripheral health systems [29, 36, 37].

### Tools

Among the various tools for malaria elimination, there was an explicit divergence of opinions on the use and efficacy of bed nets between policy makers and researchers. In contrast to policy makers, researchers thought bed nets in Southeast Asia provide little or no protection against malaria as opposed to sub-Saharan Africa where long-lasting, insecticide-treated bed nets play a leading role in malaria prevention. In Southeast Asia, because the main malaria vectors, such as *Anopheles minimus*, *Anopheles maculatus* and *Anopheles dirus,* feed outdoors during the day, specifically between 6 and 7 p.m. i.e., not when people are in bed [38, 39]. The continued distribution of bed nets may be driven by policy makers’ desire to conform with WHO guidelines for malaria vector control, which outline tools including ITNs and IRS together with supplementary interventions [40] and may be seen to provide something apparently useful to their constituencies even if bed nets, for example, offer little protection in the GMS.

Both researchers and policy makers highlighted the importance of strengthening village malaria workers and peripheral health systems. Recent evidence from a TME scale-up project along the Thai–Myanmar border showed that establishing and relying on village malaria worker network at malaria posts is critical for malaria elimination [9].

Although researchers and policy makers described the need for more effective tools for rapid elimination, opinions on the benefit of MDA were mixed [10]. MDA was seen as challenging because of the resources required, the emerging resistance against artemisinin or its partner drugs coupled with presumption that MDA could accelerate resistance [7]. However, a recent pilot study of MDA for malaria elimination in the GMS has shown that MDA was safe and could be used as a potential strategy to expedite malaria elimination as part of a package of interventions [8, 9, 41]. A potential vaccine against malaria was discussed by several researchers but none knew of an available vaccine which could be used on a large scale in the near future [42]. The feasibility of implementing successfully these tools is however dependent by the regional context where a number of challenges have been identified.

### Feasibility and challenges of malaria elimination

Researchers and policy makers explained that the region’s geography presents barriers and facilitating factors for malaria elimination [43, 44]. Highlighting the successful elimination of malaria from island nations, such as Taiwan, Vanuatu and Sri Lanka, respondents emphasized that malaria elimination from islands are likely to be more promising than in landlocked areas where malaria is more easily re-introduced [36, 43–46]. Researchers viewed peripheral health facilities as inadequately resourced for the populations they are supposed to serve in the GMS.

Researchers saw vivax malaria as a further challenge of malaria elimination [47]. In contrast to falciparum, vivax is difficult to eliminate due to the liver stages (hypnozoites) of the parasite, which can remain dormant for months to years and therefore interfere with efforts to interrupt transmission [48, 49]. In recent years, there has been a proportionate surge in prevalence of *P. vivax* infections in South East Asia [29, 48, 50–52]. For instance, in Laos, prevalence of *vivax* was 11.1% compared to 3.6% *falciparum* among a total of 888 asymptomatic persons in 2016 [50]. To achieve the goal of malaria elimination in the GMS, respondents recommended including vivax elimination as an additional consideration when developing future strategies [53, 54]. Treating vivax malaria entails providing radical therapy using 8-aminoquilonoes such as Primaquine for 14 days [55]. Primaquine however has a haemolytic effect in G6PD deficient patients and therefore a routine test for G6PD deficiency before providing treatment is essential, which adds an additional layer of challenges [53, 56].

Respondents did not specifically express concern about counterfeit antimalarials in the GMS (and their potential impact). Many have however called for the need to strengthen regional and local pharmacovigilance mechanisms to reduce the danger of further accelerating antimalarial resistance [33, 57, 58]. Considering its global and regional prevalence, concerted efforts of WHO, regional and national regulatory systems are essential to tackle counterfeit and sub-standard antimalarials [57]. Although ACTs are often freely provided at peripheral health centres, in some remote and malaria endemic regions within the GMS, the lack of health facilities, meant that patients seek treatment from illegal drug vendors and consequently receive counterfeit or sub-standard medication [28, 59]. This emphasizes need to strengthen and sustain the peripheral health workforce in the GMS.

### Funding

Securing continued funding in the face of a declining malaria burden was identified as a major challenge by many policy makers [60–62]. As malaria declines globally, researchers were worried about an increasing complacency that may overlook the need and importance for continuity in malaria control and elimination programmes [33]. Some researchers were skeptical about the actual decline of malaria, mostly because some of the data available from the national malaria reports were not completely representative of the current epidemiology and did not reflect the resurgence of malaria in specific geographic regions. This further reflects the need for a robust surveillance data for an appropriate and tailored response. Researchers were also critical of the utilization (and misuse) of funding allocated for nations embarking on malaria elimination, referring to systemic corruption [63, 64]. According to other researchers, an allocated amount of funding was misused for ineffective interventions, such as the distribution of bed nets in the GMS [38]. Some researchers described how excessive funding without adequate supervision and monitoring by funding agencies has fostered more corruption.

Challenges associated with funding are also often intertwined in the political environment of the country. Conducive political environment is indispensable for research projects to operate [10]. Political opposition at any level can affect the research projects to generate new evidence for the development of potential elimination strategies [10, 65]. A recent study showed that a lack of knowledge and misconceptions of malaria amongst policy makers has delayed implementation of MDA pilot studies in the GMS [10]. In the future, collaborations between policy makers and researchers are essential in exploring the regions’ strategies for malaria elimination.

### Future strategies

In the GMS, future strategies for malaria elimination require the tailoring of those laid out in the GTS for global malaria elimination, which includes [33]:

1. *Ensuring universal access to malaria prevention, diagnosis and treatment* A critical and foremost step would be to strengthen the peripheral health system by sustaining the network of VMWs who are at the frontline of health services in villages [21, 66]. Strengthening these networks by establishing malaria posts for diagnosis and treatment, which can expedite the access to remote and underserved populations [9]. In addition, a recent study has highlighted that collaborating with formal and informal health care providers in remote locations can further increase accessibility [28].
2. *Accelerating efforts towards elimination and attaining malaria*-*free status* Recent advances in malaria elimination strategies, such as MDA in the GMS have provided mixed results [8, 24, 41, 67]. Nevertheless, a combination of MDA together with a strengthening of the networks of village malaria workers, with the addition of malaria posts and preventive strategies has shown promising results and can be scaled up to accelerate malaria elimination [9]. In the GMS, as malaria is receding to geographically inaccessible areas, forest-goers and migrant and mobile populations, increased efforts towards these special areas and populations are indispensable to attain malaria elimination [11].
3. *Transforming malaria surveillance and response into a core intervention* Strengthening existing surveillance and response through collaboration with the health system and stakeholders should be a priority. Because malaria endemic regions in the GMS are often remote and inaccessible, surveillance and response heavily relies on the functioning of the peripheral health system [18] as well as the village malaria networks. Integrating surveillance and response into the health system, and its peripheral health structures, is impeded by weak governance, corruption and lack of qualified human resources. In light of these challenges, more health system- and operational research is critical to explore feasibility. Surveillance and response (diagnosis and treatment) through establishing malaria posts has shown promising results along the Thai–Myanmar border [9], however, its uptake by the health system and implementation at regional level are yet to be evaluated.

In addition, ongoing research in vaccine development [68], new long-lasting organophosphorous insecticides for indoor residual spraying [69], long-lasting insecticide treated bed nets (mostly for Africa) [70], new generation ACTs (or newer combinations) [71], high quality and affordable rapid diagnostic tests (RDTs) [72, 73] as well as the use of technologies such as mobile phones, global positioning system (GPS) and internet may able to optimise future interventions [73, 74].

## Strengths and limitations

Using a snowball approach to recruit participants facilitated access to difficult-to-reach policy makers involved in malaria prevention and control in the GMS as well as to key decision-makers and large international funders. Although such an approach has the potential to bias the composition of respondents, efforts were made to include experts from the field of malaria control and elimination who were recognized to have an influence in current and future malaria elimination strategies. Interviews that could not possible face-to-face were conducted via Skype/telephone or in two cases via e-mail (questionnaire). The information collected during these interviews may have been affected by the limited interaction between the interviewer and the interviewee, nevertheless, in all cases, the responses addressed the main research topics. Although not ideal, this approach provided useful information that otherwise would not have been included.

## Conclusions

Against a backdrop of increasing anti-malarial resistance and decreasing choices of anti-malarial regimens, policy makers and researchers stressed the urgency of finding new malaria elimination strategies. There was consensus that multi-pronged strategies and approaches are needed, that no single potential tool/strategy can be appropriate to all settings. Hence there is a need to customize malaria control and elimination strategies based on the better surveillance data.

Among policy makers, misunderstandings and inadequate knowledge of malaria transmission dynamics in the region contribute to support for sub-optimal interventions, delays the development of new and badly needed elimination strategies, and results in the misallocation of funds needed for other approaches. Closer collaboration between policy makers and researchers may help to overcome some of these barriers.

## Data Availability

The datasets generated during and analysed during the current study are available from the corresponding author on reasonable request.

## Appendix 1 Financial support from Global Fund and the budget for malaria elimination in the Greater Mekong Sub-region

**Table:**

Country	Received to date	Regular GF allocation (2014–2017)^a^	RAI (2014–2016)^b^	Total (2014–2017)	New allocation 2018–2020
Cambodia	$120 M (2003–)	$30 M	$15 M	$45 M	$43.0 M
Lao PDR	$54 M (2003–)	$12.5 M	$5 M	$17.5 M	$13.3 M
Myanmar	$60 M (2005–)	$26 M	$40 M	$66 M	$96.1 M
Thailand	$59 M (2004–)	$35 M	$10 M	$45 M	$23.3 M
Viet Nam	$50 M (2004–)	$7 M	$15 M	$22 M	$32.6 M
RAI inter-country			$15 M	$15 M	$34.0 M
Total (US$)	$343 M	$110.5 M	$100 M	$210.5 M	$242.3 M

Source: Global Fund

^a^Global Fund

^b^Regional artemisinin-resistance Initiative

## Abbreviations

ACT: artemisinin combined treatment
AIM: action and investment to defeat malaria
GMS: Greater Mekong Sub-region
GTS: Global Technical Strategy
G6PD: glucose-6-phosphate dehydrogenase
ITN: insecticide-treated nets
MDA: mass drug administration
RDT: rapid diagnostic test
SSI: semi-structured interview
TME: targeted malaria elimination
VMW: village malaria worker
WHO: World Health Organization
